# Comparison of peritonsillar infiltration effects of ketamine and tramadol on post tonsillectomy pain: a double-blinded randomized placebo-controlled clinical trial

**DOI:** 10.3325/cmj.2012.53.155

**Published:** 2012-04

**Authors:** Vida Ayatollahi, Shekoufeh Behdad, Maryam Hatami, Hossein Moshtaghiun, Behnam Baghianimoghadam

**Affiliations:** 1 Department of anesthesiology and critical care, Sahid Sadoughi Hospital, Shahid Sadoughi University of Medical Sciences, Yazd, Iran; 2 Department of anesthesiology and critical care, Sahid Sadoughi Hospital, Shahid Sadoughi University of Medical Sciences, Yazd, Iran; 3 Research consultant, Yazd, Iran

## Abstract

**Aim:**

To assess the effect of peritonsillar infiltration of ketamine and tramadol on post tonsillectomy pain and compare the side effects.

**Methods:**

The double-blind randomized clinical trial was performed on 126 patients aged 5-12 years who had been scheduled for elective tonsillectomy. The patients were randomly divided into 3 groups to receive either ketamine, tramadol, or placebo. They had American Society of Anesthesiologists physical status class I and II. All patients underwent the same method of anesthesia and surgical procedure. The three groups did not differ according to their age, sex, and duration of anesthesia and surgery. Post operative pain was evaluated using CHEOPS score. Other parameters such as the time to the first request for analgesic, hemodynamic elements, sedation score, nausea, vomiting, and hallucination were also assessed during 12 hours after surgery.

**Results:**

Tramadol group had significantly lower pain scores (*P* = 0.005), significantly longer time to the first request for analgesic (*P* = 0.001), significantly shorter time to the beginning of liquid regimen (*P* = 0.001), and lower hemodynamic parameters such as blood pressure (*P* = 0.001) and heart rate (*P* = 0.001) than other two groups. Ketamine group had significantly greater presence of hallucinations and negative behavior than tramadol and placebo groups. The groups did not differ significantly in the presence of nausea and vomiting.

**Conclusion:**

Preoperative peritonsillar infiltration of tramadol can decrease post-tonsillectomy pain, analgesic consumption, and the time to recovery without significant side effects.

Registration No: IRCT201103255764N2

Postoperative pain has not only a pathophysiologic impact but also affects the quality of patients’ lives. Improved pain management might therefore speed up recovery and rehabilitation and consequently decrease the time of hospitalization (1). Surgery causes tissue damage and subsequent release of biochemical agents such as prostaglandins and histamine. These agents can then stimulate nociceptors, which will send the pain message to the central nervous system to generate the sensation of pain (2-4). Neuroendocrine responses to pain can also cause hypercoagulation state and immune suppression, leading to hypoglycemia, which can delay wound healing (5).

Tonsillectomy is a common surgery in children and post-tonsillectomy pain is an important concern. Duration and severity of pain depend on the surgical technique, antibiotic and corticosteroid use, preemptive and postoperative pain management, and patient’s perception of pain (6-9). There are many studies that investigated the control of post tonsillectomy pain using different drugs such as intravenous opioids, non-steroidal anti-inflammatory drugs, steroids, ketamine, as well as peritonsillar injection of local anesthetic, opioid, and ketamine (6,7,10-14).

Ketamine is an intravenous anesthetic from phencyclidin family, which because of its antagonist effects on N methyl-D-aspartate receptors (that are involved in central pain sensitization) has regulatory influence on central sensitization and opium resistance. It can also band with mu receptors in the spinal cord and brain and cause analgesia. Ketamine can be utilized intravenously, intramuscularly, epidurally, rectally, and nasaly (15,16). Several studies have shown the effects of sub-analgesic doses of ketamine on postoperative pain and opioid consumption (7,13,15-17). Its side effects are hallucination, delirium, agitation, nausea, vomiting, airways hyper-secretion, and increased intra cerebral pressure and intra ocular pressure (10,11,15,16).

Tramadol is an opium agonist that mostly effects mu receptors, and in smaller extent kappa and sigma receptors, and like anti depressant drugs can inhibit serotonin and norepinephrine reuptake and cause analgesia (6,12,18). Its potency is 5 times lower than morphine (6,12), but it has lower risk of dependency and respiratory depression, without any reported serious toxicity (6,12). However, it has some side effects such as nausea, vomiting, dizziness, sweating, anaphylactic reactions, and increased intra-cerebral pressure. It can also lower the seizure threshold (6,12,18,19).

Several studies have confirmed the efficacy of tramadol and ketamine on post-tonsillectomy pain (6,10-12,20). In previous studies, effects of peritonsillar/ IV or IM infiltration of tramadol and ketamine were compared to each other and to placebo, and ketamine and tramadol were suggested as appropriate drugs for pain management (6,7,10-19,21). Therefore, in this study we directly compared the effect of peritonsillar infiltration of either tramadol or ketamine with each other and with placebo.

## Materials and methods

This randomized placebo-controlled clinical trial was carried out from March to September 2010 in Shahid Sadoughi Hospital, Yazd, Iran. After having received an approval from university institutional ethics committee, the study was registered in Iranian registry of clinical trials (*http://irct.ir*) as IRCT201103255764N2. Forty two patients were chosen for each group (considering the following parameters: *P* < 0.05 as significance, test power of 80%, d = 1.5 and based on previous studies S = 2). A written consent was obtained from patients' parents.

All patients were in American Society of Anesthesiologists Physical Status Classification class I and II (22), with no airway complications or systemic diseases or known psychological diseases. Reason for their surgery was recurrent or chronic tonsillitis. Patients received no medications 24 hours before surgery and had no history of sensitivity to anesthesia or any other drugs used in this study. Patients in whom the surgery had taken longer than one hour were excluded from the study. Patients received 1 mg midazolam IV plus 5 cc/kg Ringer’s Lactate solution before going to the operating room. In the operating room, all patients were monitored with pulse oximetry, ECG, capnography, and non-invasive blood pressure measurement. After injection of 1.5 μg/kg fentanyl, anesthesia was induced using 5 mg/kg thiopental sodium. Three minutes after intravenous injection of 0.5 mg/kg atracurium, the trachea was intubated. Anesthesia was maintained by N_2_O 60% and O_2_ 40%, and isoflurane 1.2%. All patients received metoclopropamide 0.2 mg/kg iv (maximum dose, 10 mg) 20 minutes before the end of the surgery. Trial drugs were coded by an anesthetic technician and were provided in 2 cc syringes. During the operation 4-5 cc/kg/h Ringer was infused.

Before surgery, block randomization was performed according to age and sex of patients. Time of anesthesia and time of surgery were determined during surgery and inter-group differences were calculated. The first group (group A) was injected 0.5 mg/kg ketamine, the second group (group B) 2 mg/kg tramadol, and the third group (group C) 2 cc NaCl (all drugs were in 2 cc volume) at the bed and anterior fold of each tonsil (1 cc for each tonsil) by an anesthesiologist who was not aware of the type of the drugs. The selected doses were chosen based on previous studies (6,7,10-19,21). The surgery was carried out by the same surgeon with sharp dissection without electrical cutter. Duration of surgery and anesthesia, systolic blood pressure (SBP), diastolic blood pressure (DBP), and heart rate were documented by an anesthetic technician who was not aware of patients’ grouping. After surgery, patients were extubated and, when they were fully awake, transferred to the recovery room, where they went under supervision of an anesthesiology resident who was not aware of the patients’ grouping. In the recovery room, hemodynamic parameters at 5, 10, 15, 30, and 60 minutes were documented and pain scores, using Children's Hospital Eastern Ontario Pain Scale (CHEOPS) (11), were recorded at 10, 20, 30, 40, and 60 minutes (Table 1). If CHEOPS score was ≥6, fentanyl 0.5 μg/kg was injected.

**Table 1:** Demographic and surgery characteristics of all patients. Age and duration of anesthesia and surgery were analyzed by ANOVA test and sex by χ^2^ test*

Variable	Ketamine	Tramadol	Placebo	*P*
Age (mean±SD, years)	8.05 ± 2.67	7.06 ± 2.21	7.40 ± 1.38	0.103
Sex (male percent)	57.10	66.70	40.02	0.061
Duration of anesthesia (mean±SD, minutes)	54.29 ± 10.96	51.67 ± 10.37	53.00 ± 14.6	0.553
Duration of surgery (mean±SD, minutes)	42.14 ± 10.43	40.56 ± 9.35	41.0 ± 12.62	0.761

*SD – standard deviation.

Following transfer to the ward, sedation and pain scores at 2, 4, 6, 8, and 12 hours were documented by an anesthesia resident using Ramsay (23) and CHEOPS scales (11). Nausea and vomiting was graded using a numeric rank scale Post Operative Nausea Vomiting (PONV) score (24). To patients who had a PONV score ≥2 metoclopramide 0.2 mg/kg was administered intravenously as a rescue antiemetic. At the recovery room, hallucinations and negative behaviors were recorded.

At the entrance to Ear, Nose, Throat (ENT) ward, 0.5cc/kg acetaminophen syrup was given. The time to the first request for analgesic and the time to first oral intake (liquid regimen) were documented. All data were analyzed by SPSS software, version 15 (SPSS, Inc., Chicago, IL, USA) using ANOVA, Bonferroni correction, and Mann-Whitney U tests.

## Results

**Figure 1:**
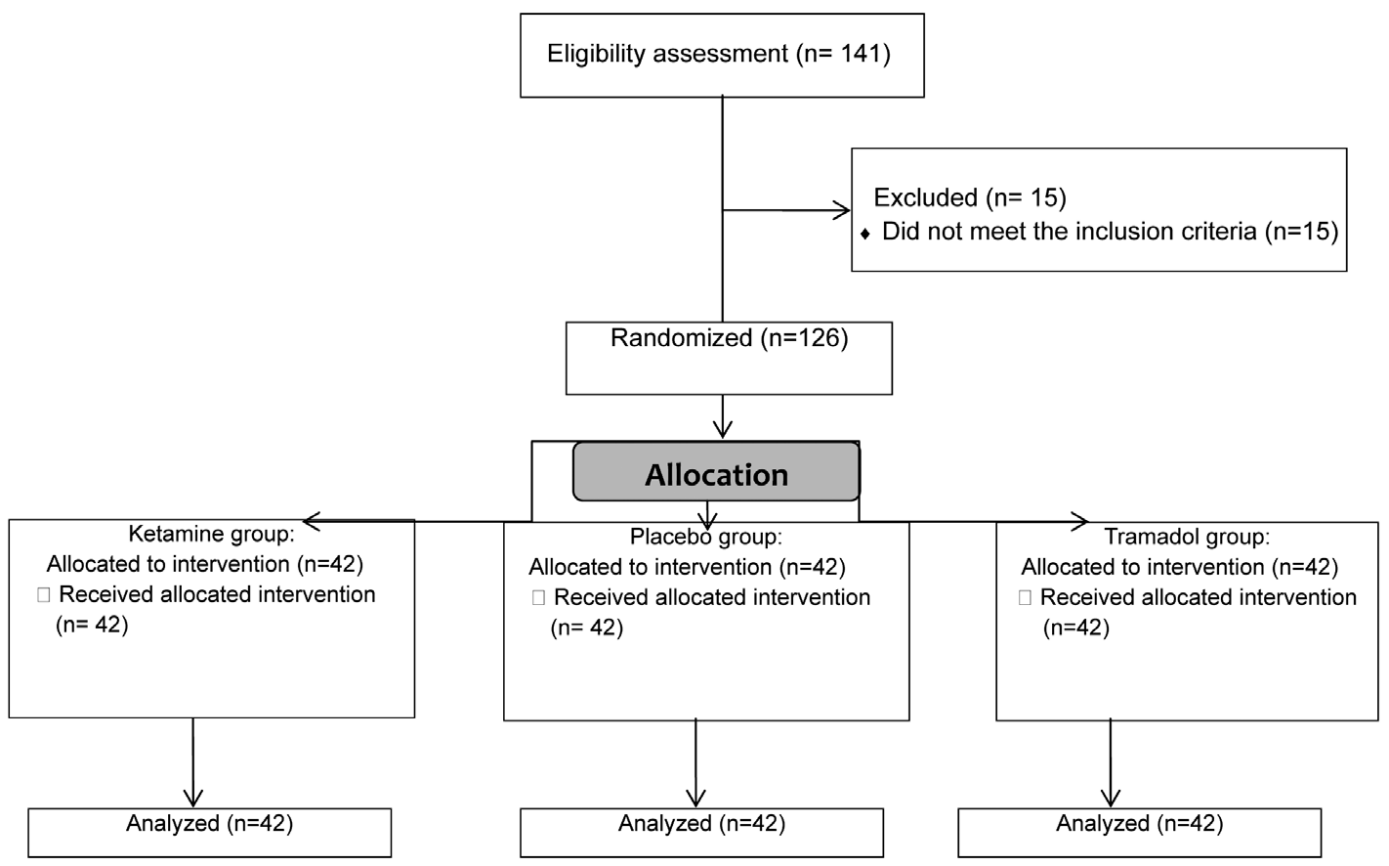
Flowchart of the study.

We compared the analgesic effect of ketamine and tramadol to placebo on 126 children between 5 and 12 years old (42 patients in each group). One hundred forty-seven patients were approached, 15 patients did not meet the inclusion criteria and were excluded from the study, and 6 were excluded due to surgery duration of more than 1 hour, which amounted to 126 patients who were finally included (Figure 1). No significant differences in age, sex, time of surgery, and duration of anesthesia were observed between the 3 groups (Table 1).

Bonferroni test showed that tramadol had significantly better pain control with shorter time to the beginning of liquid regimen and led to less demand for analgesic than placebo and ketamine group. Ketamine and placebo groups showed similar pain control (Table 2), time to the demand for analgesic (5.52 ± 2.77 hours in tramadol group vs 2.57 ± 0.84 hours in ketamine group and 2.83 ± 0.16 in placebo group, *P* = 0.035), and time to the beginning of liquid regimen (5.00 ± 1.71 hours in ketamine group vs 3.56 ± 1.72 hours in tramadol group and 5.20 ± 1.97 hours in placebo group, *P* = 0.001) (Table 3).

**Table 2:** Pain scores (mean ± standard deviations) and related *P* values at different time points after surgery

Pain (CEHOPS)^ӿ^	Placebo	*P*	Tramadol	*P*	Ketamine	*P*	Placebo
**Min 10**	5.9 ± 0.41	0.093	3.34 ± 1.02	0.003	5.79 ± 0.9	1	5.9 ± 0.41
**Min 20**	5.94 ± 0.35	0.001	3.2 ± 1.03	0.001	5.89 ± 0.9	0.15	5.94 ± 0.35
**Min 30**	6.2 ± 0.41	0.001	3.22 ± 1.53	0.001	5.93 ± 0.18	0.13	6.2 ± 0.41
**Min40**	5.6 ± 0.2	0.001	3 ± 1.34	0.001	6.2 ± 1.2	1	5.6 ± 0.2
**Min60**	5.8 ± 0.14	0.001	2.67 ± 1.4	0.001	6.02 ± 0.97	1	5.8 ± 0.14
**Hour 2**	9 ± 0.64	0.001	3.22 ± 1.77	0.001	8.57 ± 1.7	0.74	9 ± 0.64
**Hour 4**	8.40 ± 1.38	0.001	2.33 ± 1.72	0.001	7.43 ± 2.01	0.066	8.40 ± 1.38
**Hour 6**	7.60 ± 1.66	0.001	2.11 ± 1.47	0.001	6.57 ± 2.35	0.064	7.60 ± 1.66
**Hour 8**	6.6 ± 1.04	0.001	1.89 ± 1.54	0.001	7.71 ± 2	0.071	6.6 ± 1.04
**Hour 12**	5.8 ± 1.9	0.001	1.44 ± 1.27	0.001	4.43 ± 2.53	0.005	5.8 ± 1.9

*CHEOPS – Children's Hospital Eastern Ontario Pain Scale (21).

**Table 3:** Time to the beginning of liquid regimen and time of administration of the first dose of analgesic and related *P* values*

Variable	Ketamine	Tramadol	Placebo	*P*
Time to the beginning of liquid regimen (mean±SD, hours)	5.2 ± 1.97	3.56 ± 1.72	5 ± 1.71	0.001
Time to the first dose of analgesic (syrup of acetaminophen) (mean±SD, hours)	2.57 ± 0.84	5.52 ± 2.77	2.83 ± 0.16	0.035

*SD – standard deviation.

Tramadol group had significantly lower pain scores after the transfer to the recovery room or ENT ward than other two groups, but there were no significant differences between ketamine and placebo groups except in the hour 12 (*P* = 0.005) (Table 2). Five patients received additional doses of fentanyl in the recovery room, 1 in tramadol group, 2 in ketamine, and 2 in placebo groups.

There were no significant differences in SBP and DBP between groups before and during surgery. But, after surgery and during extubation tramadol group showed lower blood pressure level than other two groups (*P* = 0.001). The heart rate in tramadol group was lower than in other groups during the whole post-operation period (except 5 and 10 minutes after arriving to recovery room). There were significant differences in heart rate between tramadol and other two groups (*P* = 0.001).

The time to the first demand for analgesic was 5.2 ± 2.7 hours in tramadol, 2.8 ± 0.16 hours in ketamine, and 2.7 ± 0.08 hours in placebo group. The time to the first liquid uptake was significantly longer in tramadol group than in other groups (3.56 ± 1.70 hours in tramadol group vs 5.00 ± 1.70 hours in ketamine group and 5.20 ± 1.90 hours in placebo group) (Table 3).

Mann-Whitney U test showed that sedation scores were significantly different between ketamine and tramadol group at hours 4 (*P* = 0.005), 6 (*P* = 0.008), and 12 (*P* < 0.001), with greater scores in ketamine group, but no significant differences were observed at hours 2 (*P* = 0.146) and 8 (*P* = 0.56). Ketamine and placebo groups, except at hour 2 (*P* = 0.009), showed no significant differences in the sedation scores. Tramadol and placebo groups also showed no significant differences in sedation scores. All three groups showed no differences in sedation scores during all times, except at hour 8 (*P* = 0.845).

Prevalence of vomiting was 42.9% in ketamine group, 33.3% in tramadol group, and 38.09% in placebo group, which did not reach significance. Hallucinations were observed only in ketamine group, with the prevalence of 11.9% (*P* = 0.005).

## Discussion

This study found that peritonsillar infiltration of tramadol before tonsillectomy more efficiently decreased post-tonsillectomy pain than ketamine, with no complications. It also decreased analgesic consumption and the time to the beginning of oral liquid diet, while ketamine caused hallucinations and did not have the efficacy of tramadol in pain management and hemodynamic stabilization effects. We used 0.5 mg/kg ketamine and 2 mg/kg tramadol, which has been reported to be an effective and safe dose (10-12,15,18,19,25).

Pain following tonsillectomy is one of the most important complaints and has several unwanted consequences such as excessive use of analgesics, longer period of hospitalization, intolerance to diet, which can lead to nutritional problems, and subsequently poorer quality of life (10-12).

All analgesic methods have some potential drawbacks. Opioids can cause postoperative respiratory complications and sedation, whereas nonsteroidal anti-inflammatory drugs increase the risk of bleeding, which might require repeated surgeries to control homeostasis (6,10,12,25). Some studies reported that paracetamol could produce adequate analgesia (10), while others reported peritonsillar infiltration of opium, ketamine, and local anesthetics (6,10,11,25). Peritonsillar infiltration of local anesthetics can cause complications such as bilateral paralysis of vocal cords and severe obstruction of upper airways, acute pulmonary edema (vagus and hypoglossal block), deep neck abscesses, and brainstem stroke, which have been seen after deep infiltration and high doses of local anesthetics (7,11). Peritonsillar infiltration of opioid and ketamine did not lead to any serious complications (10-12,15,18,19,25).

We found that tramadol had significantly lower pain scores than other groups at all time points, which is in agreement with previous studies (6,12,19). Ketamine and placebo showed no differences in analgesia, which could suggest the inability of ketamine to control the pain after surgery, or a rapid absorption and wearing off of the effect. This result is not concordant with some other studies (10,11). The discrepancy could be due to higher prevalence of hallucinations and emergence reaction (11.9%) in our study than in similar studies (15,21,26,27). These reactions may have interfered with pain evaluation in the recovery room.

In our study, ketamine decreased the pain in the hour 12, which has been observed in other studies as well (10,11). A previous study concluded that low doses of intravenous injection of ketamine had no sufficient control of post tonsillectomy pain (28). The same results were observed in our study. This could be due to the usage of opioids during surgery, as opioids could mask the antagonistic effect of N-methyl-D-aspartate receptors to increase the pain to the level of placebo group, or because of the usage of ineffective doses of ketamine. Another possibility is that analgesic effect of ketamine might be delayed, so in our study the pain levels were similar in ketamine and placebo groups immediately after surgery and in the recovery room but in the ENT ward they were significantly lower in the ketamine group.

In the ward, the time to first dose of analgesic request was longer in tramadol group than in the other two groups. This value was similar in tramadol group in two other studies (6,12). Additionally, all patients in our study got acetaminophen 0.5 cc/kg in ENT ward as a preemptive analgesia (by their parents). Even with acetaminophen, the time to the first dose of another analgesic was significantly longer in tramadol group. However, in another study an opposite result was observed – 19% of patients from the placebo group, 9% from the group with IV injected ketamine, and 4% from the group with peritonsillar infiltration of ketamine required other analgesics (10).

In our study, tramadol group had significantly shorter time to the first liquid uptake than ketamine and placebo groups. These results, which are in agreement with other studies (6,12), can be explained by a lower level of pain in tramadol group.

Other studies observed no significant differences in mean arterial pressure and pain relief between ketamine and placebo groups (10,11,16,17). In fact, our finding of lower SBP and DBP in tramadol group is consistent with the finding of better analgesia. Lower BP and heart rate in tramadol than in placebo group were reported during surgery, but there were no differences in the recovery room (6). This was not in accordance with our results, since we found no differences in hemodynamic parameters between our groups during surgery but tramadol group had lower hemodynamic parameters in the recovery room. This can be due to the use of more opioids during surgery in our study or the apparent effect of tramadol in the recovery room after attenuation of opioid effect.

We also evaluated the sedation level using RAMSAY scale while patients were in the ward. In the recovery room, the scores were 3 of 5 in all groups; but by the time when the sedation scores decreased in all groups, these results were similar to other studies (6,11). It was found that the group with IV injection of ketamine had a higher level of sedation than the group with peritonsillar infiltration (7). Lower sedation effect of ketamine infiltration might be due to less systemic absorption or topical desensitization. Topical infiltrations have possibly slower, delayed, or even lower systemic absorption than IV injection, which can cause lower level of sedation in peritonsillar infiltration method.

There were no significant differences in nausea and vomiting incidence between the three groups in our study but it was higher than in similar studies (6,10). We used metoclopramide for PONV prophylaxis similar to another study (10). This study showed the prevalence of nausea and vomiting in ketamine infiltration group of 16.7%, while in our study it was 42.9%. In tramadol group in our study, the prevalence of nausea and vomiting was 33.3% but in another study it was 13% (6). This discrepancy may be due to the dehydration (since sometimes the period of “no oral uptake” before surgery took longer than 8 hours), duration of surgery more than 30 minutes (in other studies mean duration of surgery was 25 and 30 minutes respectively) (6,10), use of 60% N_2_O, and insistence of parents to start oral nutrition sooner than recommended.

Other studies found no cases of hallucinations, night terrors, or sleep disturbances caused by ketamine (10,11). However, we observed hallucinations and negative behaviors in 11.9% of our patients only in ketamine group.

We conclude that peritonsillar infiltration of tramadol before operation can decrease post tonsillectomy pain efficiently without any hemodynamic instability, sedation, or hallucinations. It can also decrease analgesic consumption and the time to the beginning of oral liquid diet, while ketamine can cause hallucinations and also does not have the efficacy of tramadol in pain management and hemodynamic stabilization effects. Therefore, we recommend the use of tramadol for management of post-tonsillectomy pain.
